# The Use of Chlorophyl in Vegetable Growth

**Published:** 1881-02

**Authors:** 


### THE USE OF CHLOROPHYL IN VEGETABLE GROWTH.

This question appears to be as yet by no means definitely settled. Pringsheim, it will be remembered, recently suggested that chlorophyl was chiefly of use as a screen to protect the subjacent cells and their contents from those rays of light which would be adverse to the secondary processes that have been distinguished as growth. But Dr. Gilbert, in his recent address to the Chemical Section of the British Association, points out that the plant may receive abundance of nitrogen, may produce abundance of chlorophyl, and be subject to the influence of sufficient light, and may yet not assimilate a due amount of carbon. He shows that the presence of a due supply of potassium salt and of sufficient available nitrogen is necessary for the proper assimilation of carbon by plants. The amount of carbon assimilated evidently does not depend on the protective power of the chlorophyl alone, nor on its chemical action. In connection with the coloring matter of leaves it has been observed that the leaves of the Virginia creeper change to the well known beautiful red hue sooner on walls exposed to the north and east, and that if the weather be wet during the time when they usually change color the red tint is only sparingly developed.—*Scientfic American.*

